# Supplementary material for: Therapeutic Potential of Latin American Medicinal Plants in Oral Diseases: From Dental Pain to Periodontal Inflammation—A Systematic Review
Source: Int J Mol Sci. 2025 Nov 27;26(23):11502. doi: 10.3390/ijms262311502 (PMC12692122; doi:10.3390/ijms262311502)
Supplement: Supplementary file 1 [file ijms-26-11502-s001.zip › ijms-3788219-supplementary.pdf]

Supplementary Material

Table S1. Complete search strategies, including search strings, date ranges, language limits, and the number of records retrieved from each database.

Database	Search string (verbatim)	Date range	Languages	Date of search	Results found
PubMed	(“medicinal plants” OR phytotherapy OR “herbal medicine”) AND (dentistry OR “oral health” OR “dental pain” OR gingivitis OR periodontitis OR “wound healing”) AND (“Latin America” OR Mexico OR Colombia OR Brazil OR Peru)	2000–2025	English, Spanish,Portuguese	March 2023 (reported retrospectively)	386
SciELO	(“plantas medicinales” OR fitoterapia OR “medicina herbolaria”) AND (odontología OR “salud oral” OR “dolor dental” OR gingivitis OR periodontitis OR cicatrización) AND (“América Latina” OR “Latinoamérica” OR Argentina OR Bolivia OR Chile OR Colombia OR Costa Rica OR Cuba OR Ecuador OR El Salvador OR Guatemala OR Honduras OR México OR Nicaragua OR Panamá OR Paraguay OR Perú OR República Dominicana OR Uruguay OR Venezuela OR Brasil)	2000–2025	Spanish, Portuguese, English	March 2023 (reported retrospectively)	25

Additional sources such as Google Scholar and regional journals (e.g., RCOE, Revista Facultad de Odontología Universidad de Antioquia, Avances en Odontoestomatología, Farmacia Profesional) were manually screened to identify relevant studies not indexed in the main databases.

Table S2. Evidence quality and study selection of included sources (n = 36).

#	Author(s), Year	Study type	Source	Evidence level	Notes
[1]	World Health Organization, 2025	Institutional fact sheet	WHO website	Grey	Global epidemiological data; not peerreviewed.
[2]	Secretaría de Salud de Medellín, 2021	Municipal health report	Government publication	Grey	Local public health report; not peerreviewed.
[3]	Valdéz Grefa & Palacios Paredes, 2022	Research article (field study)	Recimundo	Medium	Ethnobotanical field study; limited peer review, regional scope.

[4]	Fuentes, Faúndez, & Roa, 2016	Systematic review	Int. J. Odontostomatol.	High	Peer-reviewed systematic review on phytotherapy in oral mucosa.
[5]	Vara-Delgado et al., 2019	Narrative review	Rev. Arch. Med. Camagüey	Medium	Narrative review; clinical applications, limited evidence strength.
[6]	Vicente-Herrero et al., 2018	Comparative review	Rev. Soc. Esp. Dolor	Medium	Peerreviewed comparative review of scales; not primary data.
[7]	Ministerio de la Protección Social, 2008	Narrative review	Rev. Soc. Esp. Dolor	Grey	Narrative review; no original data, clinical synthesis.
[8]	Miguélez-Medrán et al., 2019	Comparative review	Rev. Soc. Esp. Dolor	Medium	Peer-reviewed comparative review of pain scales; no primary data
[9]	Rodríguez Reyes et al., 2013	Narrative review	MEDISAN	Medium	Peerreviewed Cuban journal; updated review on oral pain.
[10]	Latorre et al., 2023	Consensus statement	Neurología	High	Peerreviewed consensus statement, high-quality evidence base.
[11]	Ríos Erazo et al., 2014	Narrative review	Avances Odontoestomatol.	Medium	Peerreviewed, but narrative; limited methodological rigor.
[12]	López Sánchez & González Romero, 2001	Narrative review	Medicina Integral	Medium	Old review; peerreviewed, limited current applicability.
[13]	Servicio Canario de la Salud, 2025	Institutional health guide	Gov. of Canarias	Grey	Public health program material; not peerreviewed.
[14]	Mata Sánchez, Jiménez Méndez, & Sánchez Mendieta, 2018	Narrative review	Rev. ADM	Medium	Peerreviewed, descriptive, clinical orientation.
[15]	Iwa, 2019	Experimental study (in vitro)	Nutr. Food Sci.	High	In vitro evaluation of Moringa oleifera leaf extracts on cariogenic biofilm; peer-reviewed, strong antimicrobial evidence
[16]	Canakci & Canakci, 2007	Clinical study (observational)	J. Am. Dent. Assoc.	High	Peer-reviewed clinical study evaluating dental pain in patients receiving

					different periodontal therapies; strong clinical evidence base
[17]	Catalá Pizarro & Cortés Lillo, 2014	Narrative review	Medicina Clínica	Medium	Peerreviewed but not systematic; moderate reliability.
[18]	González-Gallego et al., 2007	Narrative review	Nutr. Hosp.	Medium	Peer-reviewed review on anti-inflammatory properties of dietary flavonoids; mechanistic synthesis without experimental data
[19]	López Silva et al., 2017	Narrative review	Med. Fam. SEMERGEN	Medium	Peerreviewed clinical review; limited methodology.
[20]	Ziegler, 2012	Narrative review	Quintessence	Medium	Peerreviewed, international dental journal.
[21]	Casariego, 2016	Narrative review	Av. Odontoestomatol.	Medium	Peerreviewed, mechanistic focus on medicinal plants.
[22]	Perazzo et al., 2004	Narrative review	J. Bras. Fitomedicina	Medium	Peerreviewed; limited methodological rigor.
[23]	Lameda Albornoz et al., 2019	Systematic review	Rev. Venezolana Inv. Odontológica	High	Peerreviewed systematic review on periodontal disease.
[24]	Lopes-Martins et al., 2002	Experimental study (in vivo)	Phytomedicine	High	Peer-reviewed pharmacological study demonstrating anti-inflammatory and analgesic effects of *Petiveria alliacea* extract in animal models; high reliability and biological validation
[25]	Santos Filho et al., 2018	Randomized clinical trial (Phase I)	Chem. Biol. Interact.	High	Clinical study testing a mucoadhesive formulation with curcuminoids and *Bidens pilosa* extract.
[26]	Criollo Jiménez, 2012	Thesis (field trial)	Univ. Nac. de Loja	Grey	Undergraduate thesis; not peerreviewed.
[27]	Rodríguez, Aguilar, & León, 2020	Narrative review	Rev. Colombiana Cienc. Hortícolas	Medium	Peer-reviewed Colombian journal; limited methodology.
[28]	Barraza et al., 2020	Bachelor’s thesis (observational/descriptive study)	Univ. Nac. San Martín	Low	Academic research describing traditional uses and knowledge of medicinal plants in

					Argentina; considered grey literature due to its thesis nature.
[29]	Zdarilová et al., 2009	In vitro experimental study	Toxicol. In Vitro	High	Demonstrated anti-inflammatory effects of *Prunella vulgaris* and rosmarinic acid on human gingival fibroblasts.
[30]	Chen et al., 2022	In vitro mechanistic study	Pharmaceuticals	High	*Rosmarinic acid* reduced LPS-induced inflammation in vascular smooth muscle cells via inhibition of MAPK/NF-κB signaling; supports its anti-inflammatory mechanism relevant to oral tissues.
[31]	Sugiharto et al., 2023	In vivo experimental study (rats)	J. Popul. Ther. Clin. Pharmacol.	High	Ethanolic extract of *Moringa oleifera* modulated the NF-κB signaling pathway promoting periodontal tissue regeneration in rats; strong in vivo evidence of anti-inflammatory and reparative effects.
[32]	Kandikur et al., 2025	Randomized clinical trial	IOSR J. Dent. Med. Sci.	High	*Moringa oleifera* mouthwash reduced plaque and gingivitis, comparable to chlorhexidine
[33]	Thuwajit et al., 2017	In vitro study	Carbohydr. Polym.	Moderate	*Acemannan* activated NF-κB and cytokine expression in gingival fibroblasts
[34]	Sánchez et al., 2020	Review article	Molecules	High	Summarizes *Aloe vera* bioactive compounds and therapeutic mechanisms
[35]	Santos et al., 2019	In vitro study	Glob. J. Med. Res. F Dis.	Moderate	*Linalool* showed immunomodulatory activity against *P. gingivalis* infection

[36]	Lozoya Legorreta, 2022	Book	Editorial CENIC	Grey	Explores Latin America's contribution to medicinal plant research

Table S3. Classification of All Cited References by Source Type (Peer-Reviewed Literature vs. Grey Literature)

N°	Abbreviated Reference	Category
[1]	World Health Organization. *Oral Health*, 2025.	Literatura gris – organismo internacional
[2]	Secretaría de Salud de Medellín. *Análisis de la Situación S. Medellín 2005–2020*, 2021.	Literatura gris – documento gubernamental
[3]	Valdéz Grefa L.K.; Palacios Paredes E.W. *Recimundo* 2022, 6(S1), 242–252.	Peer-reviewed
[4]	Fuentes F. et al. *Int. J. Odontostomatol.* 2016, 10, 539–545.	Peer-reviewed
[5]	Vara-Delgado A. et al. *Rev. Arch. Med. Camagüey* 2019, 23, 403–409.	Peer-reviewed
[6]	Vicente-Herrero M.T. et al. *Rev. Soc. Esp. Dolor* 2018, 25, 228–236.	Peer-reviewed
[7]	Ministerio de la Protección Social. *Vademécum Colombiano de Plantas Medicinales*, 2008.	Literatura gris – documento técnico
[8]	Miguélez-Medrán B.C. et al. *Rev. Soc. Esp. Dolor* 2019, 26, 233–242.	Peer-reviewed
[9]	Rodríguez Reyes O. et al. *Medisan* 2013, 17, 5080–5090.	Peer-reviewed
[10]	Latorre G. et al. *Neurología* 2023, 38(S1), S37–S52.	Peer-reviewed
[11]	Ríos Erazo M. et al. *Av. Odontoestomatol.* 2014, 30, 39–46.	Peer-reviewed
[12]	López Sánchez A.F.; González Romero E.A. *Med. Integr.* 2001, 37, 242–249.	Peer-reviewed
[13]	Servicio Canario de la Salud. *Programa de Salud Oral*, 2025.	Literatura gris – organismo estatal
[14]	Mata Sánchez N. et al. *Rev. ADM* 2018, 75, 326–333.	Peer-reviewed
[15]	Iwa S.K. *Nutr. Food Sci.* 2019, 24, 308–312.	Peer-reviewed
[16]	Canakci C.F.; Canakci V. *J. Am. Dent. Assoc.* 2007, 138, 1563–1573.	Peer-reviewed
[17]	Catalá Pizarro M.; Cortés Lillo O. *Med. Clin. (Barc.)* 2014, 143, 45–50.	Peer-reviewed
[18]	González-Gallego J. et al. *Nutr. Hosp.* 2007, 22, 287–293.	Peer-reviewed
[19]	López Silva M.C. et al. *Med. Fam. (Semer.)* 2017, 43, 141–148.	Peer-reviewed
[20]	Ziegler A. *Quintessence* 2012, 25, 8–18.	Peer-reviewed
[21]	Casariego Z.J. *Av. Odontoestomatol.* 2016, 32, 35–44.	Peer-reviewed
[22]	Perazzo F. et al. *J. Bras. Fitomed.* 2004, 2, 9–16.	Peer-reviewed
[23]	Lameda Albornoz M.A. et al. *Rev. Venez. Investig. Odontol.* 2019, 7, 85–96.	Peer-reviewed
[24]	Lopes-Martins R.A. et al. *Phytomedicine* 2002, 9, 245–248.	Peer-reviewed
[25]	Santos Filho E.X.D. et al. *Chem. Biol. Interact.* 2018, 291, 228–236.	Peer-reviewed
[26]	Criollo Jiménez M.E. *Bachelor's Thesis, Univ. Nacional de Loja,* 2012.	Literatura gris – tesis universitaria
[27]	Rodríguez M. et al. *Rev. Colomb. Cienc. Hortic.* 2020, 14, e11847.	Peer-reviewed
[28]	Barraza M.A. et al. *Bachelor's Thesis, Univ. Nac. de San Martín,* 2020.	Literatura gris – tesis universitaria

[29]	Zdarilová A. et al. *Toxicol. In Vitro* 2009, 23, 386–392.	Peer-reviewed
[30]	Chen C.-P. et al. *Pharmaceuticals* 2022, 15, 437.	Peer-reviewed
[31]	Sugiharto S. et al. *J. Popul. Ther. Clin. Pharmacol.* 2023, 30, e324–e332.	Peer-reviewed
[32]	Kandikur S. et al. *IOSR J. Dent. Med. Sci.* 2025, 24, 49–57.	Peer-reviewed
[33]	Thuwajit P. et al. *Carbohydr. Polym.* 2017, 161, 149–157.	Peer-reviewed
[34]	Sánchez M. et al. *Molecules* 2020, 25, 1324.	Peer-reviewed
[35]	Santos R.P. et al. *Glob. J. Med. Res. F Dis.* 2019, 20, 7–16.	Peer-reviewed
[36]	Lozoya Legorreta X. *Editorial CENIC,* 2022.	Literatura gris – libro institucional
[37]	Soria N. *Rev. Salud Pública Parag.* 2018, 8, 7–8.	Peer-reviewed
[38]	Congreso de Colombia. *Ley 1164 de 2007.* Diario Oficial 46.786.	Literatura gris – documento legal
[39]	Ministerio de Salud y Protección Social. *Lineamientos Técnicos…*, 2018.	Literatura gris – documento técnico
[40]	Cervantes Pérez A. *Bachelor's Thesis, UNAM,* 2017.	Literatura gris – tesis universitaria
[41]	Menon P. et al. *J. Ayurveda Integr. Med.* 2020, 12, 535–539.	Peer-reviewed
[42]	Maragliano P.; Viola T. *DentistryIQ,* 2024.	Literatura gris – fuente web profesional
[43]	Gutiérrez R.; Albarrán R. *Rev. Odontol. Los Andes* 2020, 15, 45–56.	Peer-reviewed
[44]	Ramesh A. et al. *J. Contemp. Dent. Pract.* 2018, 19, 679–683.	Peer-reviewed
[45]	Ragul P. et al. *Drug Invent. Today* 2018, 10, 637–640.	Peer-reviewed
[46]	Waizel-Bucay J.; Martín M.I. *Rev. ADM* 2007, 64, 173–186.	Peer-reviewed
[47]	Khairnar M.S. et al. *J. Indian Soc. Periodontol.* 2013, 17, 741–747.	Peer-reviewed
[48]	Nayak N. et al. *J. Clin. Exp. Dent.* 2019, 11, e1021–e1029.	Peer-reviewed
[49]	Moya Jiménez L.E. *Bachelor's Thesis, Univ. Técnica de Ambato,* 2018.	Literatura gris – tesis universitaria
[50]	Ali M.S.M.; Mohammed A.N. *J. Res. Med. Dent. Sci.* 2021, 9, 285–290.	Peer-reviewed
[51]	NIDDK. *LiverTox – Oregano*, 2023.	Literatura gris – base de datos NIH
[52]	Yuan Y. et al. *BMC Complement. Med. Ther.* 2023, 23, 61.	Peer-reviewed
[53]	Gupta R. et al. *J. Indian Soc. Periodontol.* 2014, 18, 441–445.	Peer-reviewed
[54]	El Hajj M.; Holst L. *Front. Pharmacol.* 2020, 11, 866.	Peer-reviewed
[55]	Kamath D.G. et al. *Int. J. Dent. Hyg.* 2023, 21, 211–218.	Peer-reviewed
[56]	Vairabhava L.O. et al. *BMC Complement. Med. Ther.* 2022, 22, 66.	Peer-reviewed
[57]	Hosamane M. et al. *J. Clin. Exp. Dent.* 2014, 6, e491–e496.	Peer-reviewed
[58]	Masoudi R. et al. *Res. J. Pharm. Technol.* 2024, 17, 3339–3345.	Peer-reviewed
[59]	Gupta D. et al. *J. Ayurveda Integr. Med.* 2014, 5, 109–116.	Peer-reviewed
[60]	Maurya S. *Plant Cell Biotechnol. Mol. Biol.* 2021, 22, 82–92.	Peer-reviewed
[61]	Sultana A. et al. *J. Indian Assoc. Public Health Dent.* 2024, 22, 185–190.	Peer-reviewed
[62]	Ebhohon E.; Miller D. *Int. J. Emerg. Med.* 2022, 15, 16.	Peer-reviewed
[63]	Duarte K. et al. *Eur. J. Dent.* 2022, 16, 768–774.	Peer-reviewed
[64]	Información Científica R. *Rev. Inf. Científica* 2023.	Peer-reviewed
[65]	Silva J.J. et al. *Rev. Inst. Med. Trop. São Paulo* 2014, 56, 333–340.	Peer-reviewed
[66]	Yunes R.A. et al. *J. Ethnopharmacol.* 1998, 62, 95–100.	Peer-reviewed
[67]	Lima L.D. et al. *Molecules* 2023, 28, 2546.	Peer-reviewed
[68]	Sequeda L.G. *ResearchGate DOI 10.13140/RG.2.1.3721.3047.*	Literatura gris – repositorio
[69]	Hanashiro C.T.; Miranda A.H. *J. Health Sci.* 2021, 23, 195–198.	Peer-reviewed

[70]	Millones-Gómez P.A. et al. *J. Contemp. Dent. Pract.* 2020, 21, 739–745.	Peer-reviewed
[71]	ORAS-CONHU. *Plantas Medicinales de la Subregión Andina,* 2014.	Literatura gris – organismo internacional
[72]	Agarwal A.; Chaudhary B. *J. Indian Soc. Periodontol.* 2020, 24, 354–361.	Peer-reviewed
[73]	Ramírez E.R. et al. *J. Oral Res.* 2021, 10, 275–283.	Peer-reviewed
[74]	Paucar-Rodríguez E. et al. *Rev. Cub. Investig. Bioméd.* 2021, 40(S1), e1450.	Peer-reviewed
[75]	Idir F. et al. *Front. Microbiol.* 2022, 13, 999839.	Peer-reviewed
[76]	Potra-Cicalău G.I. et al. *Med. Evol.* 2022, 28, 59–64.	Peer-reviewed
[77]	Zhao X.X. et al. *Front. Nutr.* 2021, 8, 669805.	Peer-reviewed
[78]	Kamran M.A. et al. *Medicina* 2023, 59, 1968.	Peer-reviewed
[79]	Rahmawati D.Y. et al. *Padjadjaran J. Dent.* 2023, 35, e50582.	Peer-reviewed
[80]	Lugo-Flores M.A. et al. *Biomedicines* 2021, 9, 1669.	Peer-reviewed
[81]	Das M. et al. *J. Herbmed Pharmacol.* 2023, 12, 331–336.	Peer-reviewed
[82]	Zuttion G.S. et al. *J. Dent.* 2024, 150, 105313.	Peer-reviewed
[83]	Günther M. et al. *Clin. Oral Investig.* 2022, 26, 4369–4380.	Peer-reviewed
[84]	Bayas-Morejón F. et al. *Caspian J. Environ. Sci.* 2023, 21, 123–131.	Peer-reviewed
[85]	Greene A.C. et al. *Drug Deliv. Transl. Res.* 2021, 11, 1144–1155.	Peer-reviewed
[86]	Xue Q. et al. *Front. Nutr.* 2022, 9, 1002147.	Peer-reviewed
[87]	El-Saber Batiha G. et al. *Biomolecules* 2020, 10, 202.	Peer-reviewed
[88]	Safarabadi M. et al. *Iran J. Nurs. Midwifery Res.* 2017, 22, 481–485.	Peer-reviewed
[89]	Ahmadi F. *Antibiotics* 2024, 13, 947.	Peer-reviewed
[90]	Kaboua K. et al. *Beni-Suef Univ. J. Basic Appl. Sci.* 2021, 10, 69.	Peer-reviewed
[91]	Shivananda S. et al. *J. Pure Appl. Microbiol.* 2024, 18, 476–482.	Peer-reviewed
[92]	Daga Mauricio K.M. et al. *Av. Odontoestomatol.* 2024, 40, 78–86.	Peer-reviewed
[93]	Dayal S.D. et al. *Microb. Pathog.* 2024, 197, 107033.	Peer-reviewed
[94]	Ghorbani A. et al. *Front. Dent.* 2025, 22, 10.	Peer-reviewed
[95]	Yildirim T.T. et al. *Eur. Oral Res.* 2019, 53, 99–105.	Peer-reviewed
[96]	Halboub E. et al. *BMC Oral Health* 2020, 20, 198.	Peer-reviewed

References cited in Supplementary Materials are included in the main reference list.
